# Biohydrogen production by *Chlorella vulgaris* and *Scenedesmus obliquus* immobilized cultivated in artificial wastewater under different light quality

**DOI:** 10.1186/s13568-020-01129-w

**Published:** 2020-10-27

**Authors:** Alejandro Ruiz-Marin, Yunuen Canedo-López, Paolah Chávez-Fuentes

**Affiliations:** Faculty of Chemistry, Autonomous University of Carmen, Calle 56. No.4. Av. Concordia. Col. Benito Juárez. Ciudad del Carmen, C.P. 24180 Campeche, México

**Keywords:** *Chlorella vulgaris*, *Scenedesmus obliquus*, light quality, immobilized cells, biohydrogen

## Abstract

The algal biotechnology together with the wastewater treatment can contribute to the production of renewable energies such as bioethanol, biodiesel and biohydrogen and solve many of the challenges currently facing the shortage of fossil fuels and environmental impacts. Hydrogen as the cleanest source of energy is a promising alternative to conventional fossil fuels. Among different technologies for hydrogen production, photosynthetic microorganism, such as microalgae, has a great potential to produce hydrogen, by using only water and sunlight. One of the great opportunities is that microalgae can be cultivated in urban wastewater, which contains sources of carbon and nutrients, helping to reduce the cost of biomass and energy production. Microalgae *C. vulgaris* and *S. obliquus* immobilized grown in urban wastewater was proposed for the production of biohydrogen by sulfur deprivation and two light quality prior to anaerobic condition at pH 7.5 and 30 °C and 140 µE/m^2^/s of light intensity. The results indicate that blue light induces greater algal growth than under Purple light, while the maximum hydrogen production was for cultures under purple light of 128 mL H_2_/L (productivity 204.8 mL H_2_/L/day) and 60.4 mL H_2_/L (productivity 39.18 mL H_2_/L/day) for *S. obliquus* and *C. vulgaris*, respectively. An additional advantage is the high removal of organic carbon by *S. obliquus* cultures under purple incident light compared to *C. vulgaris*, being a double benefit; energy production and wastewater treatment.

## Key points

1. Increase of the hydrogen production under manipulation of the light quality.
2. Organic carbon removal from artificial wastewater by *obliquus* and *C. vulgaris* immobilized cultures.
3. High hydrogen production (204 mL H_2_/L/day) obtained for *Scenedesmus obliquus* in cultivos with sulfur deprivation.

## Introduction

One of the great challenges for the coming decades is to obtain renewable sources that are friendly to the environment and to be able to replace the high dependence on fossil fuels. Much of the available energy is obtained from fossil energy sources; however, these are a non-renewable energy source and cause many negative impacts on the environment (Azwar et al. 2014). Therefore, several studies have been conducted to explore new sources of sustainable energy that can replace fossil fuels, and which do not have negative impacts on the environment.

One of the biofuels that has caught interest is the biodiesel obtained from microalgal culture; this can play a role as primary producer of polyunsaturated and saturated fatty acids, which can be used for biodiesel production. On the other hand, the microalgae cultures offer several additional advantages as carbon dioxide capture and fixation during photosynthesis process, as well as, the removal of nitrogen and phosphorous from wastewaters. For these reasons, the microalgae show high potential in algal biotechnology (Ruiz-Marín et al. 2010; Lim et al. 2010; De Godos et al. 2010; Wang et al. 2010; Chinnasamy et al. 2010). Another source of renewable energy produced by photosynthetic organisms is hydrogen, which contains a higher energy content of 122 kJ/g, which is 2.75 time greater than hydrocarbon fuels (Argun et al. 2008); for this reason has been investigated as a substitute for fossil fuels with a promising future, considered as an energy carrier. The first scientific investigation of H_2_ evolution by microalgae was conducted by Gaffron and Rubin (1942) who reported that microalgae *Scenedesmus obliquus* produces H_2_ in the dark at low rates and by replacing the atmosphere of the culture with nitrogen gas. Kessler (1974) concluded that hydrogen production depends on the adaptive capacity of microalgae during the transition from dark anaerobic conditions to oxygenic photosynthesis, as a means to re-oxidize the electron transport pathway.

Microalgae produces hydrogen by adopting a two-stage process (indirect biophotolysis). In stage 1, CO_2_ is fixed in the presence of sunlight through photosynthesis; that is, the microalgae produce O_2_ and accumulate carbon in the form of biomass. In stage 2, the hydrogen produced by the degradation of stored organic compounds via anaerobic takes place in the absence of oxygen using multi-enzyme systems under a series of complex biochemical reactions (Argun et al. 2009; Kapdan and Kargi 2006).

Studies have reported that the use of immobilized cells for hydrogen production is more attractive than free cells. The immobilized cells systems have advantages such as an increase in the cell retention time within bioreactors and higher metabolic activity than free cells (Tam and Wong 2000). Additional, immobilized cells help to avoid the settling during growth, phenomenon that inhibits growth due to limited gas diffusion and light penetration: therefore, immobilized cells show greater hydrogen production than free cell cultures (Rashid et al. 2013).

Several strategies have been implemented to improve hydrogen production such as the variation of light intensity, carbon source, pH, temperature and sulfur deprivation (Azwar et al. 2014; Rashid et al. 2013). The sulfur deprivation in microalgae cultures is a key factor since it inhibits protein synthesis and consequently the production of oxygen declines which is hydrogenase enzyme inhibitor (Antal and Lindblad 2005). Hydrogen production by green microalgae take place in anaerobic conditions in the dark to induce activation of enzymes involved in hydrogen metabolism. Hydrogenase sensitivity to oxygen is a big challenge for this method, so that further research is needed to develop engineered hydrogenase so that it is not sensitive to oxygen inactivation. Sulfure deprivation and anaerobic condition induce expression of hydrogenases [Fe]- in algal cells, so that continuous hydrogen production can be achieved (Ghirardi et al. 2000). [FeFe]-hydrogenase is an enzyme which plays a vital role in anaerobic metabolism, which is produce by green algae and become more efficient catalyst hydrogenases. [FeFe]-hydrogenase is able to catalysis the reversible oxidation of molecular hydrogen (Florin et al. 2001; Azwar et al. 2014).

On the other hand, hydrogen production is achieved by the degradation of internal stored compounds and can be increased by the addition of external carbon source. The nature of the carbon source and their concentration determine the economic feasibility of hydrogen production process; the use of cheaper carbon source can bring down the cost of hydrogen production significantly and an alternative is the use of urban wastewater as carbon source and nutrients (N and P) with the additional advantage that the algae culture system contributes to wastewater treatment (Ruiz-Marín et al. 2010). According to Brennan and Owende (2010), the combination of these processes will be the most plausible commercial application in the short term and a sustainable way to produce bioenergy and bio-products (Batista et al. 2015).

In general, the investigations carried out have shown that these strategies contribute to improve the hydrogen production but the method of culture using different sources of energy (light quality) and carbon source is always acknowledged to be a key factor having strong influence, however, scarce studies have been reported on the influence of the light quality (wavelength) on the hydrogen production. Chavez-Fuentes et al. (2018) reported that the light source is a variable that influences the biochemical composition, suggesting that blue light contributes to growth and purple light to lipid accumulation, so that, it is possible to regulate photosynthesis and biochemical composition by manipulating the wavelength in algal culture. The present study explores the production of hydrogen by *Chlorella vulgaris* and *Scenedesmus obliquus* immobilized cells in alginate beads as a renewable energy source, cultivated in artificial wastewater combining the effect of blue and purple light and dark anaerobic condition.

## Materials and methods

### Algal culture and acclimatization

The microalgae *Chlorella vulgaris* and *Scenedesmus obliquus* were obtained from the Biology Laboratory and Microalgae Culture Collection of the Center for Scientific Research and Higher Education of Ensenada, Baja California (CICESE), Mexico. Microalgae was cultured during acclimation in the laboratory in a sterile artificial wastewater medium, with the following concentrations (mg/L): NaCl, 7; CaCl_2_, 4; MgSO_4_.7H_2_O, 2; KH_2_PO_4_, 15 and NH_4_Cl, 115 (Ruiz-Marín et al. 2010). Trace metals and vitamins were added by following guidelines for ‘‘f/2” medium preparation (Guillard and Ryther 1962). The cultures were maintained under axenic/monospecific conditions in 250 mL flasks at 25 ± 1 °C, and at a continuous irradiance of 140 µE/m/s with fluorescent lamps (60 W) of cold white light; the photon flux rate was measured with a quantum sensor (Biospherical Instruments, QSL-100). The microalgae were transferred to fresh artificial wastewater every six days, maintaining the cultures in agitation through an orbital shaker (100 rpm).

### Preparation of immobilized algal beads

Both microalgae were firstly cultivated in an enrichment media with a nitrogen content of 90 mg NH_4_/L with the aim of increasing cell density. During exponential growth phase (determined in a particle counter Automated Cell Counter T20), an inoculum with cell density of 2 × 10^6^ cells/mL was harvested by centrifugation at 3500 rpm for 10 min. The cells were resuspended in 50 mL of distilled water to form a concentrated algal suspension with a cell density of 10 × 10^7^ cells/mL. The algal suspension was then mixed with a 4% sodium alginate solution in 1:1 volume ratio to obtain a mixture of 2% algae-alginate suspension. The mixture was transferred to a 50 mL burette and drops were formed when “titrated” into a calcium chloride solution (2%). This method produced approximately 6500 uniform algal beads of approximately 2.5 mm diameter with an initial cells number of 3.2 × 10^5^ cells/bead for every 100 mL of the algae-alginate mixture. The beads were kept for hardening in the CaCl_2_ solution for 4 hours at 25 ± 2 °C, then rinsed with sterile distilled water (Ruiz-Marín et al. 2010).

### Experimental setup and procedure

For the study of hydrogen production, the two-stage method was used, where hydrogen and oxygen synthesis occur partially separated. In the first stage, the algae growth photosynthetically under normal cultivation conditions. During the second stage, the microalgae are exposed to anaerobic conditions and sulfur is limited. With this process system, no toxic products are generated, and compounds with high added value can be produced as a result of the microalgae cultivation (Costa and De Morais 2011).

For the stage I, the immobilized cultures (*C. vulgaris* and *S. obliquus cells*) were incubated for a period of approximately 4 days with a nitrogen content of 30 mg NH_4_/L by triplicate in photobioreactors glass flasks of 1.5 L at 140 µE/m^2^/s with mechanical agitation and containing artificial wastewater culture medium at 30 °C. Each culture was provided with a light source: white, blue and purple light at 140 µE/m^2^/s.

During the experiment, the manipulation of water samples and microalgae-alginate beads was avoided to prevent contamination of the culture medium; therefore, each reactor was confined in a chamber provided with fluorescent lamps (60 W) of cold white light, blue and purple. Additionally, a hydrogen gas flow meter (Gas Flow Meter series: 32908-51 Cole-Parmer Instrument Company) was installed, connected to each photobioreactor. The experimental design and key of the treatments are shown in Table 1.

**Table 1:** Treatment design and key of each experiment for *C. vulgaris* and *S. obliquus* immobilized

Immobilized microalgae	Light source (140 µE/m^2^/s)	Key
*C. vulgaris*	White	CL_B_
	Blue	CL_A_
	Purple	CL_V_
*S. obliquus*	White	SL_B_
	Blue	SL_A_
	Purple	SL_V_

### Hydrogen production

The cultures of immobilized *C. vulgaris* and *S. obliquus* microalgae previously cultivated under white, blue and purple light for 4 days (microalgae-alginate-beads), were transferred to reactors under anaerobic conditions in sulfate free medium (replacing MgSO_4_·7H_2_O with MgCl_2_) of the following composition (mg/L): NaCl, 7; CaCl_2_, 4; MgCl_2_, 2; KH_2_PO_4_, 15; NH_4_Cl, 115 and with the addition of 10 g/L glucose (1° Brix) as a carbon source, at pH 8 and intensity of 140 µE/m^2^/s.

Each reactor was placed inside the chamber and N_2_ gas was purged into the medium for 10 min to removed dissolved oxygen. The reactor was kept under mechanical stirring for 4 days. For the measurement of hydrogen flow (mL H_2_/L) a flow meter (H_2_ Gas Flow Meter, series: 32908-51 Cole-Parmer Instrument Company) was installed for continuous recording of gas volume (mL H_2_/L) during the cultivation period.

### Glucose removal quantification

An approximate analysis of glucose consumption at the end of the incubation period under fermentation conditions for each treatment was determined from the data of soluble solids, which corresponds to the total ratio of glucose dissolved in the solution, which is represented as ° Brix in a refractometer (10 g glucose L^− 1^ equivalent to 1 ° Bx). The quantification of total reducing sugars was carried out under the method described in the Mexican standard NMX-F-312-1978.

### Statistical analyses

For statistical comparisons, the hydrogen production rate for *C. vulgaris* and *S. obliquus* cultivated in wastewater under different light intensity were analyzed by analysis of variance (ANOVA) using Statistic software (StatSoft Inc., Tulsa, OK, USA). The Tukey test (P ≤ 0.0*5*) was applied when results showed significant differences.

## Results

One alternative for the production of hydrogen by microalgae cultures is the use of cheap and available carbon sources with the aim of obtaining a profitable hydrogen production. Microalgae such as *C. vulgaris* and *S. obliquus* have shown the ability to develop in wastewater and efficiently remove nitrogen, phosphorus and organic carbon, as well as, generate high-value chemicals such as lipids that are currently being investigated for biodiesel synthesis, and have the ability to change the metabolism from autotrophic to mixotrophic or heterotrophic. For this reason, currently urban wastewater has been considered as a profitable culture medium, providing nutrients and carbon sources to sustain the growth of *C. vulgaris* and *S. obliquus*.

In the present study, during the first stage of photosynthetic growth, both immobilized microalgae showed no inhibitory effects on growth in relation to the quality of incident light (white, blue and purple). A cell count at the end of the culture period showed that during stage 1, microalgae *C. vulgaris* increased the number of cells from 3.0 × 10^5^ cells/beads to 14 × 10^5^ for white light, 17 × 10^5^ under blue light, and 9 × 10^5^ cells/beads under purple light, while for *S. obliquus*, the increase in cell density was in the order from 3.0 × 10^5^ cells/beads to 8 × 10^5^, 10 × 10^5^, and 7 × 10^5^ cells/beads for white, blue and purple light, respectively.

**Fig. 1:**
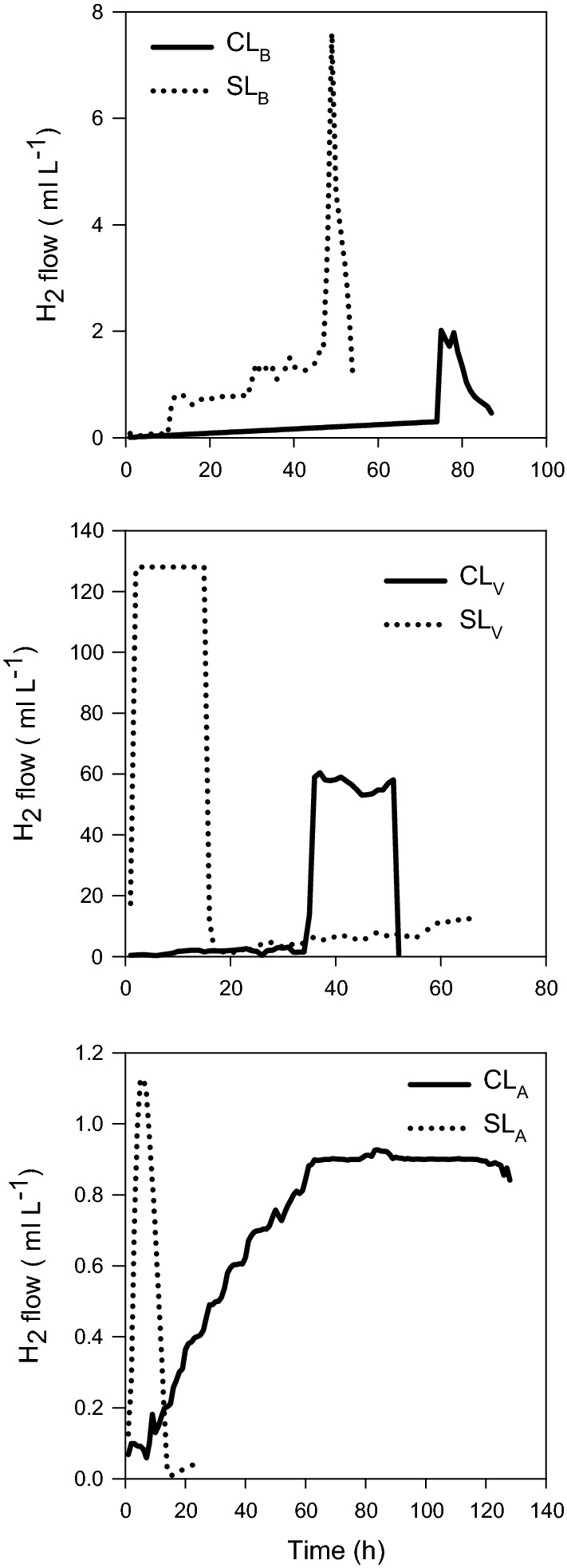
H_2_ production by *C. vulgaris* and *S. obliquus* immobilized under different wavelength (white, blue and purple light)

During the second stage (anaerobic conditions), the hydrogen production was measured until the maximum production was observed. During the cultivation period the hydrogen production by *C. vulgaris* and *S. obliquus* immobilized cells was proportional to the glucose consumption (Table 2). The glucose uptake showed significant differences (p = 0.001); for the cultures under purple light was observed a high glucose removed of 70% and 90% for *C. vulgaris* and *S. obliquus* immobilized cells, respectively (Table 2). This was related with the maximum production of H_2_ for both microalgae, where the high hydrogen production was for *S. obliquus* was of 128 mL H_2_/L (productivity of 204.8 mL/H_2_/L/day); while for *C. vulgaris* a peak of maximum production was of 60.4 mL H_2_/L (productivity of 39.18 mL H_2_/L/day) (Fig. 1; Table 2).

**Table 2:** Removal of glucose in dark-anaerobic and maximum productivity of H_2_ in cultures of *C. vulgaris* and *S. obliquus* immobilized in alginate beads (data are shown as mean ± SD, n = 3)

Microalgae	Key	Glucose removed (g/L)	Maximum production H_2_ (mL/L)	Productivity (mL H_2_/L/day)
*C. vulgaris*	CL_B_	from 10 to 6	2.02	0.65^a^ ± 0.25
	CL_A_	from 10 to 8	0.91	0.265^a^ ± 0.19
	CL_V_	from 10 to 3	60.4	39.18^b^ ± 23.1
*S. obliquus*	SL_B_	from 10 to 5	7.6	3.72^c^ ± 1.6
	SL_A_	from 10 to 9	1.13	4.52^c^ ± 2.7
	SL_V_	from 10 to 0.5	128.0	204.8^d^ ± 61.2

Means followed by similar letters showed not significantly different (Tukey; p ≤ 0.05)

Both immobilized microalgae grown under anaerobic conditions showed the ability to change their metabolism and use sources of organic carbon (glucose) for growth. This is an opportunity to carry out integral microalgae cultures in wastewater, achieving significant energy savings in wastewater treatment systems and obtaining chemical products of high commercial value (Table 2). According to the results obtained, microalgae *S. obliquus* grown in urban wastewater is proposed as a candidate for the production of hydrogen and to be able to participate in wastewater treatment systems using organic carbon sources.

## Discussion

In the present study, the maximum increase in cell density for the cultures under blue light for both immobilized microalgae were similar to the reported by Chavez-Fuentes et al. (2018) concluding that free cells cultures of *C. vulgaris* and *S. obliquus* exposed to blue light favored growth, while purple light induces lipid accumulation (% w/w). Other studies suggest that blue light contains energy more efficient for carrying out photosynthesis (Das et al. 2011; Korbee et al. 2005). While the purple light due to high energy that can emit, cause effects on the growth of *C. vulgaris* and *S. obliquus* (Mohsenpour et al.  2012). Although the available information is scarce on growth in immobilized systems under different light sources, it has been documented that in free cell cultures growth changes can occur when going from a phototrophic to a mixotrophic culture system in microalgae, as reported by Canedo-López et al. (2016) in mixotrophic cultures (white light/dark) of *Chlorella vulgaris* showed a low cell density in artificial wastewater medium and urban wastewater of 11.65 × 10^6^ cells/mL and 10.76 × 10^6^ cells/mL, respectively; compared with phototrophic culture of 17.66 × 10^6^ cells/mL and 15.26 × 10^6^ cells/mL, respectively. Concluding that lighting conditions (continuous light/photoperiods) influence algal growth. On the other hand, Papazi et al. (2012) reported a lower mixotrophic growth of *Scenedesmus obliquus* for 5 days with dichlorophenol from 4.5 × 10^5^ cells/mL to 11.9–16.1 × 10^5^ cells/mL; with the aim of increasing the rate of hydrogen production. Although the comparison is not absolutely correct between free and immobilized cells, because the conditions and the parameters used in the literature are totally different, it is a fact that mixotropic conditions tend to decrease cell density compared to phototrophic cultures.

In addition to the above, the mixotrophic culture under different light sources could also cause changes in algal growth such as those reported by Chavez-Fuentes et al. (2018) suggesting that the intensity and light source modifies the growth and biochemical composition, reporting the highest concentrations of biomass dry weight (g/L) and cellular density (cells/mL) for a white light source (140 µE/m^2^/s) of 0.3 g/L and 4.9 × 10^6^ cells/mL, respectively and, for blue light of 0.4 g/L and 4.5 × 106 cells/mL, respectively; in contrast to the observed for purple light (0.23 g/L and 2.97 × 10^6^ cells/mL, respectively) and yellow light (0.12 g/L and 3.13 × 10^6^ cells/mL, respectively). This is congruent with the reported in the present study, cultivation of immobilized cells showed a low cell density (cell/beads) under purple light with respect to blue light, suggesting the light quality is a factor key that can modify the growth and, consequently, algal biochemical composition in cultures with artificial wastewater.

In a fact that the immobilization of cells on substrates offers a greater advantage over free cells in suspension, since the immobilized cellular matter occupies less space, requires a smaller volume of growth medium, is easier to handle, and can be used repeatedly for products generation. In addition to photosynthetic bacteria, immobilized green algal cultures has also been employed to increase the yield and efficiency of H_2_ production in these eukaryotic oxygenic photosynthesis systems. Immobilized systems have been found to be more efficient at switching between the oxygenic photosynthesis (growth) and the hydrogen production modes. Kosourov and Seibert (2009) reported for *C. reinhardtii* immobilized on alginate films in sulfur/phosphorus-deprived cultures, a high cell density (2000 µg Chl/mL) and hydrogen production rates (12.5 µmol/mg Chl/h). It is a fact that immobilization helps to improve the hypoxic environment in the vicinity of the cells, thus promoting conditions for H_2_-production and making more efficient use of the carbon sources contained in the culture media.

During the second stage, hydrogen production by *C. vulgaris* and *S. obliquus* immobilized cells was proportional to the glucose consumption (Table 2). These suggested that the maximum glucose uptake for the cultures of *C. vulgaris* and *S. obliquus* (70% and 90%, respectively) under purple light (Table 2) were related with the maximum production of H_2_. The ability to remove organic carbon has been reported in numerous microalgae in mixotrophic culture systems making this attractive for use in wastewater treatment systems. Canedo-López et al. (2016) reported a similar removal of total organic carbon (TOC) for *Chlorella vulgaris* in mixotrophic free culture in artificial wastewater (70.5−86.0%) and urban wastewater (43.7−56.2%). Other studies in free cell culture suggest a high removal of chemical oxygen demand (COD) for *Chlorella *sp. and *Scenedesmus obliquus* from 63 to 88% (Lu et al. 2016; Gupta and Pawar 2018). This suggests that both microalgae can change its metabolism from autotrophic to mixotrophic according to prevailing conditions, and continue to use inorganic and organic carbon (Ogbonna and Tanaka 2000; Liang et al. 2009; Mandal and Mallick 2011).

The high hydrogen production obtained for *S. obliquus* of 128 mL H_2_/L (productivity of 204.8 mL H_2_/L/day) and for *C. vulgaris* of 60.4 mL H_2_/L (productivity of 39.18 mL H_2_/L/day) (Fig. 1; Table 2) were high to the reported by Chader et al. (2009) for *Chlorella sorokiniana* of 1.35 mL H_2_/L/h in free cell cultures, containing acetate as the only carbon source under optimal conditions of pH: 7.2 and light intensity of 120 µE/m^2^/s at 30 °C. Rashid et al. (2013) evaluated the production of hydrogen by immobilized *C. vulgaris* optimizing parameters such as: pH, carbon source (glucose, fructose, sucrose and malt extract) and light intensity. The authors reported a maximum production of 812, 874, 1315 and 1144 mL/L for the different carbon sources at pH 8, respectively. These values were high compared to that obtained in the present study, but other factors could intervene in the production of H_2_ when microalgae are cultivated in wastewater, such as organic load, carbon sources and competition and predation by other microorganisms. According to Das and Veziroglu (2001) the high concentration of carbon source modifies the metabolic pathway and leads to production of unwanted by-products and, because of this, it is important to consider each of these factors during hydrogen production.

In cultures of *C. vulgaris* under white, purple and blue light a prolonged lag phase was observed before hydrogen production of 70 h, 35 h and 10 h, respectively, suggesting this time as required to change the metabolism from autotrophic to heterotrophic to use the available carbon sources in the wastewater and be able to express the hydrogenase enzyme for subsequent hydrogen production. In contrast, *S. obliquus* only presented a lag phase in cultures under white light (Fig. 1), compared with the cultures under purple and blue light suggesting a high capacity of the microalgae to adapt under these cultivation conditions and, to activate the enzyme hydrogenase for production of hydrogen in the first hours of dark anaerobic condition. In fact, microalgae *C. vulgaris* showed an insufficient ability to degrade glucose into protons, and consequently, during this period of prolong time lag, the hydrogenase enzyme was not active sufficiently to convert them into hydrogen.

Although the biochemistry of immobilized cells was not determined in the present study, some considerations may be mentioned. It is likely that a light source with a high level of energy (purple light) induces lower growth but with a high uptake of organic carbon and potentiate the production of hydrogen, while in the case of blue light (low energy level) induces growth but lower hydrogen production during the anaerobic stage. In this context, the results could suggest that the accumulation of lipids which is induced by light quality (purple light) contributes to a better the use of external carbon sources, since microalgal cells under these conditions will have a lower growth, content of chlorophyll and carbohydrates-proteins, so it forces a metabolic change for a better use of external carbon sources and quickly activates the hydrogenase enzyme under anaerobic conditions, which could be related to low energy uptake from purple light for the phtosynthesis, compared to those cultures under white and blue light where the carbohydrate and protein content assumes that are high. However, more studies should be carried out to know which of these two conditions during anaerobic dark phase cultivation contributes to increased hydrogen production. In fact, microalgae *S. obliquus* represents a better proposal for the hydrogen production than *C. vulgaris* and is a candidate for the wastewater treatment with the ability to efficiently remove the carbon source from urban wastewater and obtain bio-hydrogen as an energy source.

## Data Availability

All data generated or analyzed during this study are included in this published article.
